# Sex Allocation in Relation to Host Races in the Brood-Parasitic Common Cuckoo (*Cuculus canorus*)

**DOI:** 10.1371/journal.pone.0036884

**Published:** 2012-05-15

**Authors:** Frode Fossøy, Arne Moksnes, Eivin Røskaft, Anton Antonov, Andrzej Dyrcz, Csaba Moskat, Peter S. Ranke, Jarkko Rutila, Johan R. Vikan, Bård G. Stokke

**Affiliations:** 1 Department of Biology, Norwegian University of Science and Technology (NTNU), Trondheim, Norway; 2 Department of Avian Ecology, University of Wroclaw, Wroclaw, Poland; 3 Biological Institute, Eötvös Lorand University, Budapest, Hungary; 4 Department of Biology, University of Eastern Finland, Joensuu, Finland; Institut Pluridisciplinaire Hubert Curien, France

## Abstract

Sex allocation theory and empirical evidence both suggest that natural selection should favour maternal control of offspring sex ratio in relation to their ability to invest in the offspring. Generalist parasites constitute a particularly interesting group to test this theory as different females commonly utilize different host species showing large variation in provisioning ability. The common cuckoo (*Cuculus canorus*) is a generalist brood parasite that lays its eggs in the nest of many different passerine birds, but each female tends to specialize on one particular host species giving rise to highly specialized host races. The different host species show large variation in their ability to invest in the parasitic offspring, presenting an opportunity for female cuckoos to bias offspring sex ratio in relation to host species quality. Here, we investigate host-race specific sex allocation controlling for maternal identity in the common cuckoo. We found no evidence of any significant relationship between host race and sex ratio in one sympatric population harbouring three different host races, or in a total of five geographically separated populations. There was also no significant association between host quality, as determined by species-specific female host body mass, and cuckoo sex ratio. Finally, we found no significant relationship between individual cuckoo maternal quality, as determined by her egg volume, and sex ratio within each host race. We conclude that the generalist brood-parasitic common cuckoo show no significant sex-ratio bias in relation to host race and discuss this finding in light of gene flow and host adaptations.

## Introduction

Fisher [1] showed theoretically that a 1∶1 sex ratio should be evolutionary stable as there otherwise would be a frequency-dependent advantage to the rarer sex. Later on, Hamilton [2] showed that females being able to control offspring sex towards the rarer one should be selected for by natural selection and Trivers and Willard [3] suggested that natural selection should favour maternal control of offspring sex ratio in relation to their ability to invest in the offspring. Trivers and Willard [3] argued that in polygynous species displaying sexual size dimorphism, male reproductive success is highly dependent on size and shows a higher variance than for females. Thus, males in good condition will outreproduce their equally good condition sisters, whereas females in poor condition will do better than their equally poor condition brothers. Hence, females in good condition will benefit by producing high-quality sons over high-quality daughters, whereas females in poor condition will benefit by producing average-quality daughters over average-quality sons. In support of this theory, several empirical studies have found an association between maternal condition at conception and sex ratio of their offspring in vertebrates, particularly in ungulates [4], [5], [6].

In birds, females are the heterogametic sex and the sex determining division in avian meiosis occurs prior to ovulation and fertilization, providing the females with an unusual direct opportunity to modify offspring sex ratio [7]. Although the exact mechanism remains elusive [7], [8], many studies have found that larger, high-quality females produce relatively more sons than smaller females of inferior quality also in birds [9], [10], [11], [12].

The avian brood-parasitic common cuckoo (*Cuculus canorus*) therefore constitutes a particular interesting species for investigating sex allocation. The common cuckoo is highly polygynous with ca. half of all males siring offspring with more than one female and is sexually size dimorphic [13], [14], [15]. This generalist parasite lays its eggs in the nest of many different passerine birds but each female tends to specialize on one particular host species giving rise to highly specialized host races often mimicking the eggs of their specific host species in both colour and size [16], [17], [18]. The offspring of different cuckoo races are thus raised by different host species showing large differences in host quality. Cross-fostering of cuckoo nestlings have found that larger hosts produce faster growing nestlings and larger fledglings compared with smaller hosts [19]. Moreover, different cuckoo host races vary in egg size, demonstrating size mimicry with their specific host species [18]. Since fledgling mass is a good indicator of adult body mass [20] and egg size is related to body size [21], it is reasonable to suppose that cuckoo races parasitizing larger host species are larger than cuckoo races parasitizing smaller host species. According to sex allocation theory we should therefore expect that cuckoo races utilizing large high-quality host species show a male-biased sex ratio whereas cuckoo races utilizing smaller host species show a female-biased sex ratio. Large males from high-quality hosts will outreproduce their sisters, and natural selection will thus select for females being able to skew offspring sex ratio towards sons in the large host races. However, this assumes that large cuckoo males mate across host races and gain access to females from smaller host races. Genetic data suggest that approximately forty percent of cuckoo males sire offspring in more than one host species and thus evidently mate across host races [13], [14]. We are therefore most likely to find evidence for the Trivers-Willard hypothesis in populations where several cuckoo races breed together within one locality (i.e. in sympatry).

A few previous studies have not revealed any significant relationship between host species and sex ratio in the common cuckoo or the brood-parasitic brown-headed cowbird (*Molothrus ater*) [22], [23], [24]. However, so far no one has controlled for maternal identity in their analyses, potentially causing a problem of pseudoreplication. The cowbird offspring is raised alongside the host nestlings and therefore have to compete for food [25]. As for the cuckoo, males show a higher growth rate and fledging mass than females but in contrast to the cuckoo two different patterns of sex ratio bias could be expected. The larger cowbird males may easier outcompete the larger hosts than the smaller cowbird females and thus male-biased sex ratio in the larger host would be expected. On the other hand, males are the more costly sex and would grow faster in competition with smaller host nestlings, thus suggesting a male-biased sex ratio in the smaller host [24].

Here, we investigate host-specific sex-ratio in the common cuckoo using molecularly determined sex of cuckoo offspring found in nests of different host species. Firstly, we use multiple genetic markers to determine sibship among the cuckoo offspring in order to control for maternal identity in the statistical analyses. Secondly, we analyse sex of cuckoo offspring found in one sympatric population harbouring three different host races, where gene flow is known to occur [13]. Thirdly, we combine the data from the sympatric population with data from four other localities spread out in Europe, of which three populations contain only one host race (i.e. allopatric) and where gene flow is likely to be restricted. In line with sex allocation theory, we expect that host races exploiting larger host species show a more male-biased sex ratio than host races utilizing smaller host species, and that this pattern should be most evident in the sympatric populations.

## Materials and Methods

### Ethics statement

Collection of DNA complied with the legal regulations of each country and every possible step was taken to minimize any potential harm to the birds. Cuckoo nestlings were gently lifted from the nest and blood (5–25 µl) was drawn by puncturing either the brachical or femoral vein. Permits for working with cuckoos and their hosts and collect DNA samples was received in each country; permits were issued by the Ministry of Environment and Water in Bulgaria; the Municipal Office in Hodonin (3C2KA/2003) in the Czech Republic; the Southeast Finland Regional Environment Centre in Finland; the Middle-Danube-Valley Inspectorate for Environmental Protection (31873), Nature Conservation and Water in Hungary; and the Regional Ethical Committee in Wrocław and the Faculty of Biology (KWB.0118.1-2003), University of Wrocław in Poland.

### Data collection

Data on three sympatrically breeding cuckoo host races parasitizing corn buntings (*Milaria calandra*), great reed warblers (*Acrocephalus arundinaceus*) and marsh warblers (*A. palustris*) was collected in the surroundings of Zlatia, north-western Bulgaria [13], [18]. Additional data were collected from one sympatrically breeding cuckoo population parasitizing reed warblers (*A. scirpaceus*) in Luzice, Czech Republic where also great reed warblers, marsh warblers and sedge warblers (*A. schoenobaenus*) are parsitized [26]. Data on three allopatrically breeding cuckoo populations were collected in eastern Karelia, Finland, parasitizing redstarts (*Phoenicurus phoenicurus*) [27], Apaj, Hungary, parasitizing great reed warblers [28] and Milicz, Poland, parasitizing reed warblers [29] (Table 1). DNA was collected either through blood samples of cuckoo nestling or tissue samples from dissecting ejected/unhatched eggs. The genetic samples were preserved in 96% ethanol for subsequent analyses.

**Table 1:** Sex ratio of cuckoo offspring in relation to population and host species.

Country	Locality	Host species	Population ID	Body mass* (g)	Sympatry†	No mothers^‡^	Mean no offspring (range)	No offspring	No females	No males	Sex ratio^§^
Bulgaria	Zlatia	Corn bunting	BGR-CB	39.6	Sympatric	15	2.2 (1–7)	33	19	14	0.42
Bulgaria	Zlatia	Marsh warbler	BGR-MW	11.4	Sympatric	11	2.1 (1–7)	23	13	10	0.43
Bulgaria	Zlatia	Great reed warbler	BGR-GRW	28.4	Sympatric	18	1.8 (1–6)	33	16	17	0.51
Hungary	Apaj	Great reed warbler	HUN-GRW	28.4	Allopatric	13	1.8 (1–4)	23	14	9	0.39
Czech Republic	Luzice	Reed warbler	CZE-RW	11.8	Sympatric	6	2.5 (1–6)	15	7	8	0.53
Poland	Milicz	Reed warbler	POL-RW	11.8	Allopatric	13	1.5 (1–3)	19	9	10	0.53
Finland	North Karelia	Redstart	FIN-RS	15.0	Allopatric	17	1.9 (1–4)	32	13	19	0.59

* Female host species-specific body mass [41].

† Sympatric with other cuckoo host races ^‡^Sibship and number of mothers inferred by genetic markers ^§^ Sex ratio uncorrected for maternal identity.

### Genetic analyses

DNA was extracted from the blood/tissue samples using E.Z.N.A. blood DNA kit (Omega Bio-Tek Inc, Norcross, USA). All loci were amplified by polymerase chain reaction (PCR) on a GeneAmp PCR System 9700 (Applied Biosystems, Foster City, USA) and run on a 3130XL Genetic Analyser (Applied Biosystems, Foster City, USA). The sex marker and microsatellites were scored in Genemapper v. 3.7 (Applied Biosystems, Foster City, USA), and the mitochondrial sequence data were assembled and manually checked in Geneious v. 4.7.6 [30]. To ensure consistency, all genotypes were scored by one person (FF).

We used the CHD1-M5 primer in combination with P8 for molecular sex determination [31], [32]. Genetic samples from two adult males and one adult female were used to confirm the validity of sex determination in the common cuckoo.

### Statistical analyses

Many cuckoo nestlings were collected within the same geographic area and could be either full- or halfsiblings, and hence potentially represent a problem of pseudoreplication. We therefore analysed a genetic dataset on 13 microsatellite markers (Ccμ01, Ccμ09, Ccμ13 [33]; Ccμ60, Ccμ88, Ccμ100, Ccμ108, Ccμ119, Ccμ137 [34]; Cba08, Clu02, Clu03 and Clu05 [35]; see [13] for details on each marker) using the software Colony [36] to determine family relationships. In contrast to most other similar software that only considers pair-wise comparisons, Colony utilizes a full-pedigree likelihood approach, which considers the likelihood of the entire pedigree structure and allows for the simultaneous inference of parentage and sibship. Moreover, Colony allows the user to add information on known relationships among the offspring to increase the probability of correctly assigning sibship. We therefore added information on geographic locality, mitochondrial haplotype and egg appearance to exclude sibling relationships. Two offspring sampled from two different localities are not likely to share either the same father or mother. Moreover, two offspring having different mitochondrial haplotypes are not likely to share the same mother. And finally, two offspring having identical mitochondrial haplotypes but originating from different looking eggs are also not likely to share the same mother. The appearance of individual eggs from each cuckoo female is highly repeatable and can be used to assign individual eggs to different females, although different females may produce similar eggs [37]. For the grouping of mitochondrial haplotypes, we utilized a dataset on 411 bp of the left-hand hypervariable control region (CCRL1A: 5′-CATGATACATTACATGTATGCCTG-3′ and CCRH1: 5′CTGAAATAGTATGAATGTATCTGTG-3′ [38]). Only offspring showing a probability of 0.95 or higher of being either a fullsibling or halfsibling to one or a group of offspring were included in the analyses, and in total eight offspring of known sex were therefore conservatively excluded.

Binominal mixed models were used to test for host-specific differences in sex-ratio throughout. Maternal identity was always included as a random factor to control for maternal sibship in the analyses. The analyses were performed in R v2.13.0 [39] using the lme4 package v0.999375–39 [40].

## Results

We successfully analysed sex and sibship of 89 cuckoo offspring originating from 44 different females in the sympatric population of Zlatia and its surroundings. Among these offspring, 41 were males and 48 were females showing an overall sex ratio of 0.46. There were no significant differences in sex ratio between any pair of the three cuckoo host races (Table 1, Table 2). We also tested whether individual maternal quality was associated with sex ratio variation *within* each host race. We ran a second model where we included host race identity in addition to maternal identity as a random factor. As we lack information on individual cuckoo maternal condition or quality, we used egg volume as a proxy. Egg volume is related to adult body size [21] and therefore reflect female condition. However, egg volume was not significantly associated with sex (estimate ± SE = 0.82±0.80, z = 1.02, p = 0.31, N = 71 offspring, 38 mothers). In addition, we also ran separate models for each host race and locality, controlling for female identity, but none of the tests revealed any significant relationship between cuckoo egg volume (i.e. female body size) and sex (all p>0.13).

**Table 2:** Pair-wise comparisons of sex ratio in relation to population and host species using binomial mixed models controlling for female identity.

	BGR-CB				BGR-MW				BGR-GRW				HUN-GRW				CZE-RW				POL-RW			
	Estimate	±	SE	*P*	Estimate	±	SE	*P*	Estimate	±	SE	*P*	Estimate	±	SE	*P*	Estimate	±	SE	*P*	Estimate	±	SE	*P*
BGR-MW	0.12	±	0.55	*0.82*																				
BGR-GRW	0.31	±	0.49	*0.53*	−0.18	±	0.55	*0.74*																
HUN-GRW	−0.14	±	0.55	*0.81*	−0.26	±	0.60	*0.67*	−0.44	±	0.55	*0.42*												
CZE-RW	0.44	±	0.63	*0.48*	0.32	±	0.67	*0.64*	0.13	±	0.62	*0.83*	−0.58	±	0.67	*0.39*								
POL-RW	0.41	±	0.58	*0.48*	0.29	±	0.63	*0.65*	0.11	±	0.57	*0.85*	0.55	±	0.63	*0.38*	−0.03	±	0.69	*0.97*				
FIN-RS	0.68	±	0.50	*0.17*	0.56	±	0.56	*0.32*	0.38	±	0.50	*0.45*	−0.82	±	0.56	*0.14*	0.25	±	0.63	*0.70*	−0.27	±	0.58	*0.64*

See table 1 for explanation of population names.

We found no significant differences between any pair of the five geographically separated populations (all p>0.14, N = 178 offspring, 93 mothers). Furthermore, there were no significant differences in sex ratio between any pair of the five host races pooled across the different geographic localities (all p>0.17, N = 178 offspring, 93 mothers), or between sympatric and allopatric populations, including host species as a random effect to control for host race identity (estimate ± SE = −0.17±0.30, z = −0.56, p = 0.58, N = 178 offspring, 93 mothers).

**Figure 1:**
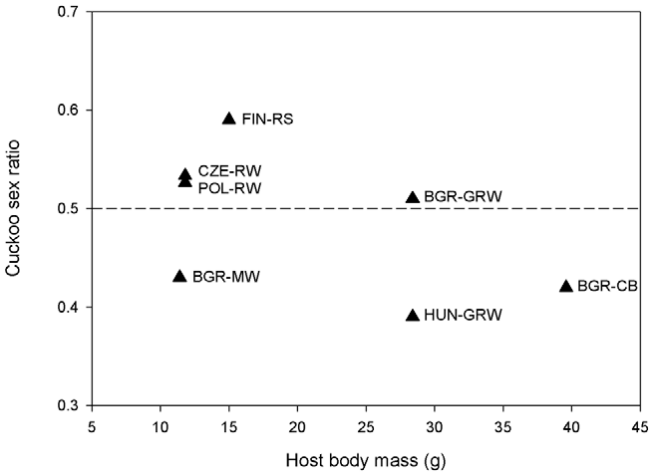
The relationship between host quality, represented by female species-specific host body mass, and cuckoo sex ratio (uncorrected for maternal identity). Values above the dashed line indicate a male-biased sex ratio. See Table 1 for explanation of population names.

In order to analyze the effect of host species quality we included species-specific host female body mass [41] as a covariate in the mixed model. However, female host body mass were not significantly associated with cuckoo sex ratio (estimate ± SE = −0.016±0.014, z = −1.12, p = 0.27, N = 178 offspring, 93 mothers, 5 species, Figure 1). Moreover, the slope was negative, and hence opposite of what we predicted.

## Discussion

In this study, we investigated host-specific sex ratio in the generalist brood-parasitic common cuckoo. We hypothesized that sex ratio of cuckoo offspring should vary in relation to host species quality in accordance with predictions from sex allocation theory. However, we found no evidence of any significant relationship between host race and sex ratio in one sympatric population harbouring three different host races, or in a total of five different geographically separated populations. Hence, our results corroborate the few previous studies on avian brood parasites [22], [23], [24]. There was also no significant association between host quality, as determined by female species-specific host body mass and cuckoo sex ratio. Furthermore, we found no significant difference in sex ratio between cuckoo host races breeding in sympatry or allopatry with other host races. Finally, we found no significant relationship between cuckoo individual maternal quality, as determined by her egg volume, and sex ratio within each host race. Thus, the common cuckoo does not seem to bias offspring sex ratio in relation to either host species quality or individual maternal quality.

According to theory, female cuckoos have both the ability and opportunity to increase their own fitness by selectively producing the rarer sex. Firstly, avian females have an unusual direct opportunity to modify offspring sex ratio because the sex determining division in avian meiosis occurs prior to ovulation and fertilization [7] and several empirical studies provide evidence that female birds do bias the sex ratio of their offspring in relation to body size [9], [10], [11], [12]. Secondly, different host species vary greatly in parental quality which affects growth rate and fledgling body mass of the nestling cuckoo [19] and which thus most likely also results in differential adult body size among cuckoo host races. Sex bias in relation to host quality has been demonstrated in haplo-diploid parasitoid wasps that oviposits their eggs in immobilized fly pupae; female wasps produce more daughters when utilizing larger high quality host pupae than when they utilize smaller host pupae, increasing offspring production by two to three percent [42], [43]. Strangely, cuckoo females do not seem to utilize this valuable opportunity. However, we have to stress that the number of individuals as well as host populations in our sample may not be sufficient to draw unequivocal conclusions. Also, having population-specific measures of host quality rather than general species-specific values would be preferable, as host species will vary in size across populations. However, the slope of the non-significant relationship between host quality and cuckoo sex ratio was negative and opposite of our prediction.

The traditional Trivers-Willard hypothesis rests on three assumptions. Firstly, parental condition must be associated with offspring condition; secondly, any difference in offspring condition must persist into adulthood; and thirdly, condition must differentially affect the mating success of one sex more than the other [3], [6]. Unfortunately, we lack empirical data to unequivocally confirm whether these assumptions are met in the common cuckoo. However, current data suggests that cuckoos utilizing larger species have larger eggs, faster growing offspring and heavier fledglings than cuckoos utilizing smaller species [18], [19]. Whether these differences only reflect differential parental abilities between the host races or also contains a genetic component among host races is unknown. However, we recently demonstrated a small but significant genetic differentiation both at mitochondrial and autosomal loci between three allopatric host races indicating that a genetic component may exist [13].

The occurrence of genetic differentiation and assortative mating may explain why females show no evidence of sex ratio bias in relation to host quality. If males commonly mate within their own host race, males from large host races will not compete directly with males from smaller host races, and therefore not achieve a higher reproductive success than their sisters. Hence, natural selection will not select for a male-biased sex ratio *sensu* Trivers and Willard [3]. We have recently suggested that the occurrence of assortative mating in combination with empirical evidence from other avian species makes it unlikely that the functional loci controlling eggshell colouration is found on the female-specific W-chromosome in the common cuckoo as previously assumed [13]. If this is correct, males mating across host races would provide their own daughters with a disadvantage by possibly disrupting the egg mimicry to its own host species. This would select against cuckoo males mating across host races and may therefore counteract any selective benefit provided by a differential sex ratio investment in relation to host quality.

A comparative study on the family Cuculidae suggests that sexual size dimorphism has more likely evolved via coevolution than sexual selection in this taxa [44]. The theory behind this is that smaller females producing smaller eggs have a selective advantage in the coevolutionary arms race. Although cuckoos have the smallest eggs in relation to their body size [21], their eggs are usually larger than those of their hosts. Hence, smaller eggs laid by smaller cuckoo females would look more similar in size to the hosts own eggs, and face a lower risk of being rejected. Therefore, the advantage of sex ratio bias in relation to male condition *sensu* Trivers-Willard may be counteracted by the selection for smaller females in cuckoos. This could also explain the lack of any relationship between sex ratio and female quality, as determined by cuckoo egg volume, within each host race. It is also possible that egg volume does not capture maternal quality in the way we expect, and that other measures like investment in hormones and anti-oxidants in the oocyte would be more suitable [45], [46], [47].

We conclude that the generalist brood-parasitic common cuckoo show no evidence of sex-ratio bias in relation to host race, host race quality or individual maternal quality.
